# Advances in Perovskite Light-Emitting Diodes Possessing Improved Lifetime

**DOI:** 10.3390/nano11010103

**Published:** 2021-01-04

**Authors:** Peng Xiao, Yicong Yu, Junyang Cheng, Yonglong Chen, Shengjin Yuan, Jianwen Chen, Jian Yuan, Baiquan Liu

**Affiliations:** 1 Guangdong-Hong Kong-Macao Intelligent Micro-Nano Optoelectronic Technology Joint Laboratory, Foshan University, Foshan 528225, China; xiaopeng@fosu.edu.cn (P.X.); cheng-jy@outlook.com (J.C.); c19875916364@163.com (Y.C.); ysj981006@163.com (S.Y.); iamjwen@126.com (J.C.); yuanjian@fosu.edu.cn (J.Y.); 2 School of Electronics and Information Technology, Sun Yat-sen University, Guangzhou 510275, China

**Keywords:** light-emitting diode, perovskite, lifetime, light outcoupling, encapsulation

## Abstract

Recently, perovskite light-emitting diodes (PeLEDs) are seeing an increasing academic and industrial interest with a potential for a broad range of technologies including display, lighting, and signaling. The maximum external quantum efficiency of PeLEDs can overtake 20% nowadays, however, the lifetime of PeLEDs is still far from the demand of practical applications. In this review, state-of-the-art concepts to improve the lifetime of PeLEDs are comprehensively summarized from the perspective of the design of perovskite emitting materials, the innovation of device engineering, the manipulation of optical effects, and the introduction of advanced encapsulations. First, the fundamental concepts determining the lifetime of PeLEDs are presented. Then, the strategies to improve the lifetime of both organic-inorganic hybrid and all-inorganic PeLEDs are highlighted. Particularly, the approaches to manage optical effects and encapsulations for the improved lifetime, which are negligibly studied in PeLEDs, are discussed based on the related concepts of organic LEDs and Cd-based quantum-dot LEDs, which is beneficial to insightfully understand the lifetime of PeLEDs. At last, the challenges and opportunities to further enhance the lifetime of PeLEDs are introduced.

## 1. Introduction

As an emerging energy-saving technology, perovskite light-emitting diodes (PeLEDs) have been demonstrated to be highly promising for a wide range of applications (e.g., display, lighting, and signaling) owing to their impressive merits including high efficiency, superior color purity, high luminance, low operational voltage, and low power consumption [1,2,3,4,5]. In 2014, Friend and coworkers reported the first room temperature organic-inorganic hybrid CH_3_NH_3_PbBr_3_ PeLED, achieving a maximum external quantum efficiency (EQE) of 0.1% and luminance of 364 cd m^−2^ [6]. In 2015, Zeng and coworkers realized all-inorganic CsPbX_3_ PeLEDs, obtaining a maximum EQE of 0.12% and luminance of 946 cd m^−2^ [7]. In 2016, Demir and coworkers demonstrated the first bright FAPbBr_3_ PeLED, obtaining a maximum current efficiency (CE) of 6.4 cd A^−1^ and luminance of 2714 cd m^−2^ [8]. Since then, a large number of efforts have been made to improve the performance of PeLEDs [9,10,11].

Currently, the maximum EQE of both organic-inorganic hybrid and all-inorganic PeLEDs can exceed 20%, which is comparable to that of state-of-the-art organic LEDs (OLEDs) and Cd-based quantum-dot LEDs (QD-LEDs) [12,13,14,15,16,17]. The full width at half-maximum (FWHM) of electroluminescence (EL) spectrum in PeLEDs can be as narrow as <20 nm, which is superior to that of advanced OLEDs and Cd-based QD-LEDs [18]. In addition, the maximum luminance of PeLEDs can surmount 591,197 cd m^−2^ [19]. As a comparison, the maximum luminance of Cd-based QD-LEDs is 614,000 cd m^−2^ [20] while the maximum luminance of OLEDs is generally below 200,000 cd m^−2^ [21,22,23,24]. Furthermore, the turn-on voltage of PeLEDs can be as low as <2 V, which is even lower than the value of the corresponding optical bandgap [25,26,27]. Therefore, these outstanding properties suggest the huge potential of PeLEDs for practical applications. Nevertheless, the longest lifetime of PeLEDs is only ~250 h at 100 cd m^−2^ [28], which is far away from the real commercialization (e.g., an essential demand of 100,000 h at 100 cd m^−2^ for displays [29,30,31]). Thus, the stability issue becomes the most challenging hindrance for PeLEDs to be commercialized.

Fortunately, increasing endeavors have been taken to improve the lifetime of PeLEDs via the design of perovskite emitting materials and the innovation of device engineering. For example, the stability of PeLEDs will be improved if defects in perovskite emitting materials are passivated (e.g., the use of 5-aminovaleric acid to passivate defects) [32,33,34]. The device stability will be also enhanced if good charge balance is accomplished (e.g., the improvement of electron injection to balance the hole injection) [35,36,37]. On the other hand, despite optical effects (e.g., light outcoupling) and advanced encapsulations have been scarcely investigated to improve the lifetime of PeLEDs, these two factors are believed to be significant to decide the stability according to the evolution of OLEDs and Cd-based QD-LEDs [38,39,40,41,42,43]. Hence, by virtue of the related concepts about optical effects and advanced encapsulations in OLEDs and Cd-based QD-LEDs, it is conducive to loosen the stability bottleneck of PeLEDs.

Herein, state-of-the-art concepts to improve the lifetime of PeLEDs will be comprehensively reviewed from the perspective of four important factors (i.e., perovskite emitting materials, device engineering, optical effects, and advanced encapsulations). First, the fundamental concepts determining the lifetime of PeLEDs will be presented. Second, the strategies to improve the lifetime of PeLEDs will be highlighted. In particular, the two factors of optical effects and encapsulations that are rarely investigated in PeLEDs for the improved lifetime will be discussed based on the related concepts of OLEDs and Cd-based QD-LEDs, which is expected to deeply understand the stability of PeLEDs. Finally, the issues and ways to further enhance the lifetime of PeLEDs will be clarified. 

## 2. Fundamental Concepts Determining the Lifetime of PeLEDs

### 2.1. Perovskite Emitting Materials

Over the past several years, both organic-inorganic hybrid and all-inorganic perovskites have been intensively investigated to be efficient emitting materials of LEDs for the sake of high photoluminescence quantum yield (PLQY), pure color emission, affordable cost, and readily tunable emission by adjusting over anion identity and/or perovskite size [44,45,46]. Perovskites usually have formula ABX_3_, where A-site is [CH_3_NH_3_]^+^ (MA^+^) and [CH(NH_2_)_2_]^+^ (FA^+^) for hybrid perovskites or Cs^+^ for all-inorganic perovskites, B-site is mostly Pb^2+^, and X-site is Cl, Br, I or mixed halide systems (Cl/Br, Br/I) [47,48,49]. To date, both ABX_3_ colloidal nanocrystals having different morphologies (e.g., nanowires, nanoplatelets, and QDs (nanospheres/nanocubes)) and ABX_3_ thin films obtained through vacuum-evaporating or spin-coating the precursors have been demonstrated to be elegant emitting materials [50,51,52].

**Figure 1:**
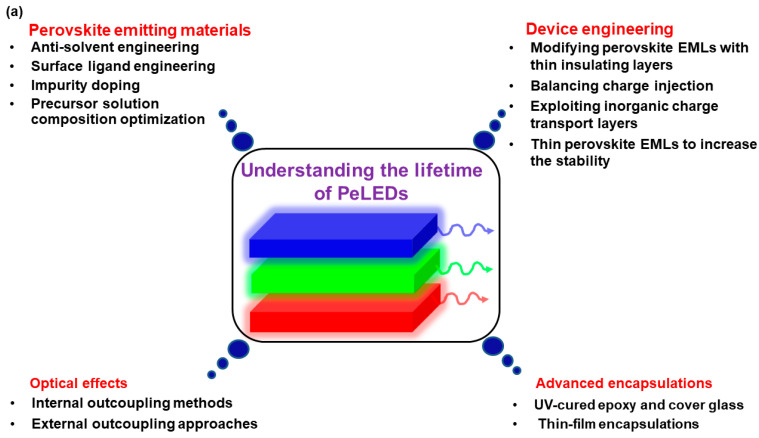

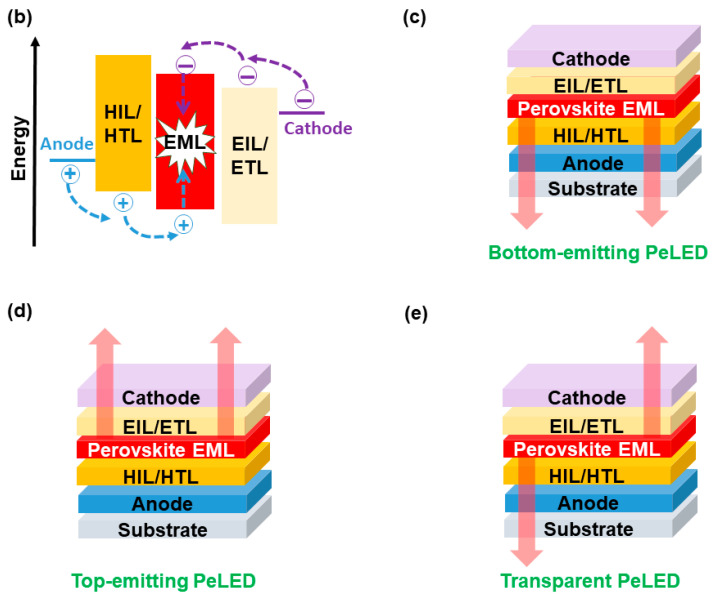
(**a**) Schematic diagram summarizing the main factors to determine the lifetime of perovskite light-emitting diodes (PeLEDs) and corresponding strategies to improve the stability. (**b**) Schematic illustration describing the working mechanism of PeLEDs. (**c**) Device architecture of bottom-emitting PeLEDs. (**d**) Device architecture of top-emitting PeLEDs. (**e**) Device architecture of transparent PeLEDs.

Despite continuous endeavors enable perovskites achieving huge success in various technologies (e.g., solar cells, lasers, and photodetectors), the instability nature of perovskites restricts the further applications [53,54,55]. In terms of PeLEDs, it is essential to deeply understand the device lifetime before commercialization (Figure 1a). As the emitting materials of PeLEDs, perovskites can affect the device lifetime due to the following four main factors [56,57,58,59,60,61,62,63]. (i) If polar solvents are used, perovskites will be generally inefficient (e.g., losing ligands, optical characteristics, as well as structural integrity) because of the inherent ionic nature. Therefore, both the solvent of perovskites and the solvent of nearby charge transport materials have an influence on the stability of PeLEDs. For example, an annealing process is required for the typical electron transport layer (ETL) zinc oxide (ZnO) to ensure the lifetime of PeLEDs, since ZnO is usually dissolved in polar solvents [64,65,66]. (ii) Perovskites are easily affected by external stresses (e.g., electric field, heating, light, oxygen, and moisture) because of the low formation energy. Particularly, the halide segregation can occur under the electric field, deteriorating the device lifetime and efficiency due to the formation of trap centers. Additionally, the electric field can cause the ion migration, leading to trap states in perovskite emitting layers (EMLs) and/or mobile ions being diffused into charge transport layers/electrodes, which is harmful to the stability. (iii) The film morphology of perovskite EMLs plays a critical role in the performance of solution-processed PeLEDs, since the leakage current will take place if EML films are too rough. (iv) The trade-off between surface passivation and charge injection through ligands control is significant to the lifetime of PeLEDs, since ligands (e.g., oleic acid (OA) and oleylamine (OLA)) possess double-side effect: enough ligands ensure high PLQY and ink stability by eliminating defects, while excessive ligands prevent the charge injection due to the formation of insulating layers as OA and OLA exhibit poor electric conductivity. Furthermore, it is worthy noted that perovskite emitting materials in the blue region are obviously unstable compared to other-color realms [67,68,69], which is similar to the situation of OLEDs and Cd-based QD-LEDs [70,71,72].

### 2.2. Device Engineering 

If perovskite emitting materials are set, the lifetime of PeLEDs is highly sensitive to device engineering. The emission mechanism of PeLEDs can be concluded as follows, as shown in Figure 1b [73,74,75,76]. First, charges (i.e., holes and electrons) will be injected from electrodes (i.e., anode and cathode) upon connecting power sources. Then, holes and electrons will reach the EML through hole injection layer (HIL)/hole transport layer (HTL) and electron injection layer (EIL)/ETL, respectively. Excitons will be formed for radiative recombination if holes and electrons meet at the EMLs, resulting in the intentional emissions. For the EL process, nonradiative decays (e.g., Auger recombination) are required to be averted. Therefore, device engineering (e.g., charge injection, transport, accumulation, leakage, balance, and recombination) plays a crucial role in the performance of PeLEDs [77,78,79,80]. 

Device engineering can affect the lifetime because of the following three main factors. (i) The stability of PeLEDs is vulnerable to the stability of charge transport materials [81]. Hence, stable charge transport materials are required. For example, the typical HTL 4,4-*N*,*N*-dicarbazolebiphenyl (CBP) is not only prone to crystallization due to a low glass transition temperature (62 °C) but also electrochemically decomposed to act as nonradiative recombination centers and/or luminance quenchers during device operation, decreasing the stability [82]. Recently, Liu et al. achieved 4.5-fold longer lifetime in colloidal LEDs by replacing CBP with relatively stable HTL *N*,*N*′-di-(1-naphthyl)-*N*,*N*′-diphenyl-(1,1′-biphenyl)-4,4′-diamine (NPB)/4,4′,4″-tris(carbazol-9-yl)-triphenylamine (TCTA) [83]. (ii) The stability of PeLEDs is strongly associated with charge balance [84,85,86]. This is because excess electrons or holes can easily cause emitters charging, resulting in poor efficiency and stability. (iii) The stability of PeLEDs is related to the interface engineering between the perovskite EMLs and charge transport layers. For instance, charge leakage occurs if the charge blocking ability of charge transport layers is weak, while charge accumulation takes place if a large charge barrier exists between EMLs and charge transport layers [87,88,89,90].

### 2.3. Optical Effects

The maximum internal quantum efficiency (IQE) of PeLEDs is supposed to be 100%, whereas the EQE (*η*_ext_) is decided by the outcoupling factor (*η_out_*), the fraction of excitons that can potentially radiatively decay (*r*), PLQY (*q*), and charge balance (*γ*), which is written as [83]:

(1) $$\eta_{ext}=\eta_{out}\times r\times q\times \gamma$$

The maximum EQE of PeLEDs is 20%, if an outcoupling factor of 0.2 is used according to the classical ray optics theory (i.e., Snell’s law). Hence, high EQE can be achieved via the enhancement of the light extraction efficiency. For example, Tang et al. obtained CsPbBr_3_ PeLEDs with a maximum EQE of 28.2% through using bioinspired moth-eye nanostructures at the front electrode/perovskite interface together with a half-ball lens to enhance the outcoupling efficiency [91].

On the other hand, the manipulation of optical effect plays a vital role in the lifetime of PeLEDs, since excess thermal energy is inevitably generated by the trapped light. The wave-guiding effect is originated from the mismatch of refractive index (N) among perovskites (N ≈ 2.3), organic layers (N ≈ 1.6–1.76), transparent electrodes (e.g., N ≈ 1.8–2.2 for indium tin oxide (ITO)), glass substrate (N ≈ 1.5) and air (N ≈ 1.0), most photons generated by exciton recombination are trapped via the total internal reflection at interfaces inside PeLEDs [92,93,94,95]. For typical ITO-based OLEDs, light undergoes four modes [96,97,98,99,100]: (i) external mode (≈20%), (ii) substrate mode, where the light is trapped at the glass/air interface (optical loss ≈ 30%); (iii) ITO/organic mode, where the light is trapped at the ITO/substrate interface (optical loss ≈ 40%); and (iv) surface plasmon-polariton (SPP) mode, which is associated with metallic electrode/organic interface (optical loss ≈ 10%). In the case of PeLEDs, another more total internal reflection exists at the interface of perovskite EML/organic charge transport layer. Therefore, the thermal energy generated at interfaces is readily conducted to perovskite EMLs and charge transport layers, degrading the stability of PeLEDs.

### 2.4. Advanced Encapsulations

Since perovskites and charge transport materials are vulnerable to the environmental stress, advanced encapsulations are essential to guarantee the lifetime of PeLEDs. Especially since flexible electronics and transparent electronics are becoming more and more important to our life, flexible encapsulations are urgently required to satisfy the increasing demand of various kinds of applications [101,102,103]. Fortunately, the current encapsulation technology of PeLEDs can take from the experience of OLEDs and Cd-based QD-LEDs. In general, the utilization of ultraviolet (UV)-cured epoxy and cover glass along with a desiccant inside devices is the most common approach for the encapsulation of bottom-emitting PeLEDs (Figure 1c). On the other side, although the top-emitting PeLED (Figure 1d) or transparent PeLED (Figure 1e) has been negligibly reported until now, the thin-film encapsulation technique is believed to be widely used for these types of PeLEDs in the near future according to the development of top-emitting or transparent OLEDs and Cd-based QD-LEDs [104,105,106]. In addition, thin-film encapsulations are also broadly adopted in flexible PeLEDs. In order to lower the water vapor transmission rate of the encapsulation to the ideal levels (10^−6^ g m^−2^ day^−1^) [107], thin-film encapsulations of are required to satisfy the requirements including high transmission efficiency (>90%) in the visible region, low-stress materials for enough thick layers, low processing temperatures (<130 °C), pinhole-free coating being densely packed, continuous as well as highly conformal, and minimized UV light or sputtering damage.

## 3. Strategies to Obtain PeLEDs with Improved Lifetime

### 3.1. Basic Aspects of the Lifetime of PeLEDs

According to the above-mentioned concepts, perovskite emitting materials, device engineering, optical effects, and advanced encapsulations are four critical factors to determine the lifetime of PeLEDs. Aside from EQE, CE, power efficiency (PE), luminance, and driving voltage, lifetime is a key parameter to judge whether PeLEDs can be commercialized. In general, T_50_, which represents the time when the luminance decays to its half value, is used to characterize the lifetime of soft-materials based LEDs [108,109,110]. To be more precise, T_95_, T_90_, and T_80_, are also widely utilized in the practical applications. In 1996, Tang et al. first proposed an equation to evaluate the lifetime of OLEDs [111]:

(2) $$L_{0}\times t_{1/2}=C$$

where *L*_0_ is the initial luminance, $t_{1/2}$ is the half-life and *C* is a constant. Since then, other equations have also been proposed to describe the lifetime and most of them indicate that higher *L*_0_ result in shorter $t_{1/2}$. Nowadays, the most commonly utilized equation to characterize the lifetime is [112,113,114]:

(3) $$L_{0}{}^{n}\times t_{1/2}=C$$

where *n* is the acceleration coefficient and relies on materials set, device architectures, and emission colors [115,116,117,118,119]. Toward the real commercialization, PeLEDs should satisfy the essential demand of 100,000 h at 100 cd m^−2^ for displays or 10,000 h at 1000 cd m^−2^ for lighting. To date, despite that no PeLED can possess a long lifetime, a large number of efforts have been made to explore the improvement of device stability, which will be described in the following sections.

### 3.2. The Design of Perovskite Emitting Materials 

Perovskite emitting materials play a vital role in the lifetime of PeLEDs. Generally, all-inorganic perovskites show superior thermal stability to organic-inorganic hybrid perovskites. As a result, the longest lifetime of red, green, and blue PeLEDs are reported based on all-inorganic perovskites. With the step-by-step understanding of perovskite emitting materials, the stability of PeLEDs has been gradually improved. Specifically, four main strategies are used to design perovskite emitting materials (i.e., anti-solvent engineering, surface ligand engineering, impurity doping, and precursor solution composition optimization), which have been demonstrated to effectively enhance the device stability.

#### 3.2.1. Anti-Solvent Engineering 

To pursue high-performance optoelectronic devices, the morphology of perovskite films should be well controlled. Currently, anti-solvent engineering is a broadly utilized strategy to achieve high-quality pinhole-free perovskite films [120,121,122]. With such strategy, it is highly promising to enhance the stability of PeLEDs. In particular, the use of anti-solvent engineering on all-inorganic halide perovskites is effective to extend the device lifetime.

**Figure 2:**
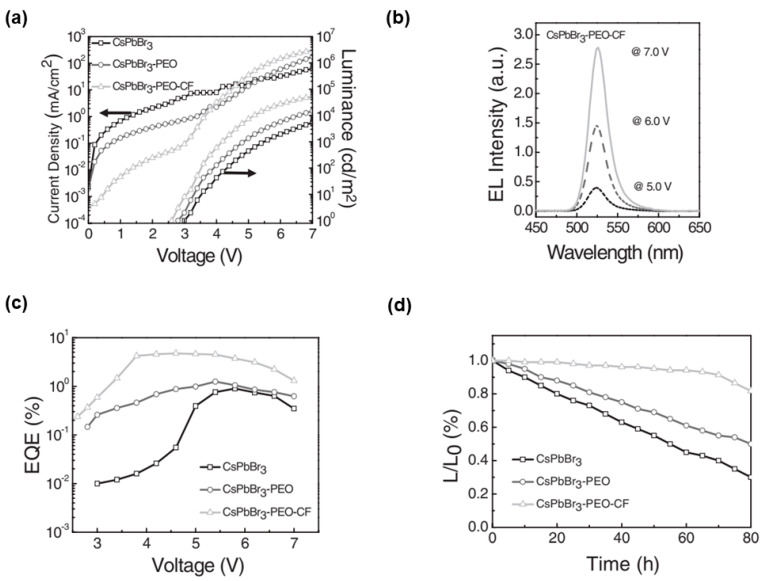
(**a**) Current density and luminance. (**b**) EL spectra of CsPbBr_3_-PEO-CF film-based device. (**c**) External quantum efficiency (EQE). (**d**) Stability of PeLEDs based on pristine CsPbBr_3_, CsPbBr_3_-PEO, and CsPbBr_3_-PEO-CF. Reproduced from reference [124]. John Wiley and Sons, 2017.

The short PL decay lifetime (i.e., the fast free exciton emission decay) of perovskites means severe nonradiative energy transfer to trap states, while small crystal sizes generate more trap states at adjacent grain boundaries to deteriorate the PLQY [123] To achieve uniform and pin-hole free perovskite films with low trap state densities (i.e., low defects by controlling their grain sizes), Sun et al. embedded perovskite microcrystals in a dielectric polymer matrix polyethylene oxide (PEO) and used an anti-solvent vapor method to further increase CsPbBr_3_ crystal size in the early stage of crystal growth [124]. Additionally, with anti-solvent of chloroform (CsPbBr_3_-PEO-CF) post-treatment, CsPbBr_3_ thin films exhibited remarkable improvement in both PL efficiency and lifetime. PeLEDs were developed with the device architecture of ITO/PEDOT:PSS/perovskites/TPBi/LiF/Al, where perovskites were pristine CsPbBr_3_, CsPbBr_3_ embedded in PEO (CsPbBr_3_-PEO), and CsPbBr_3_-PEO-CF. As shown in Figure 2, the optimized PeLEDs with CsPbBr_3_-PEO-CF showed a maximum EQE of 4.76% and excellent long-term stability under ambient conditions. For example, CsPbBr_3_-PEO-CF PeLEDs maintained 82% of its initial efficiency after 80 h continuous operation in ambient air (relative humidity ≈ 60%), which was much more stable than CsPbBr_3_ and CsPbBr_3_-PEO PeLEDs. The enhanced stability was attributed to the surface passivation by PEO, large grain sizes and fewer grain boundaries (e.g., perovskite microcrystals with a size of ≈5 µm along the in-plane direction with active emitting composite of 90%).

Since the common anti-solvents (e.g., chloroform, chlorobenzene, toluene) are hazardous, green anti-solvents are more beneficial to lower the pollution and facilitate the commercial applications of PeLEDs [125]. Several efficient green anti-solvents (e.g., ethyl acetate [126,127,128], methoxybenzene [129], and isopropyl alcohol [130]) have been reported to increase the grain size with a reduction in grain boundary related defects, resulting in optoelectronic devices with improved performance. Especially, Xu et al. made a systematic comparison (e.g., structural, morphological, and optoelectronic properties) between green and traditional toxic anti-solvents on MAPbBr_3_ and demonstrated that perovskite films engineered with ethyl acetate were superior to the counterparts engineered with chlorobenzene and toluene, boosting the performances of the corresponding PeLEDs due to the reduced defects as well as the improved film morphology and crystallinity [131].

#### 3.2.2. Surface Ligand Engineering 

The regulation of surface state of perovskite nanocrystals is critical to the performance of PeLEDs, since ligands can not only passivate the surface to get rid of defects but block charge injection [132]. Additionally, perovskites are relatively ionic and sensitive to many polar solvents, indicating that the PLQY and ink stability of perovskites can be greatly affected by ligands [133]. For example, Zeng et al. used a recyclable treatment on perovskites with hexane/ethyl acetate mixed solvent to balance surface passivation and charge injection, achieving a 50-fold EQE improvement (up to 6.27%) via ligand density control [134]. Then, they reported a room-temperature triple-ligand surface engineering strategy to play the synergistic role of short ligands of tetraoctylammonium bromide (TOAB), didodecyldimethylammonium bromide (DDAB), and octanoic acid (OTAc) toward perovskites with a high PLQY of >90%, unity radiative decay in its intrinsic channel, stable ink, and effective charge injection/transportation, leading to PeLEDs with a peak EQE of 11.6% and PE of 44.65 lm W^−1^, which were the most efficient PeLEDs with colloidal CsPbBr_3_ QDs at that time [135]. Bakr et al. developed a postsynthesis passivation process by using a bidentate ligand (i.e., 2,2′-iminodibenzoic acid) for the high chemical and phase stabilities of CsPbI_3_ nanocrystals, obtaining red PeLEDs with a maximum EQE of 5.02% [136].

**Figure 3:**
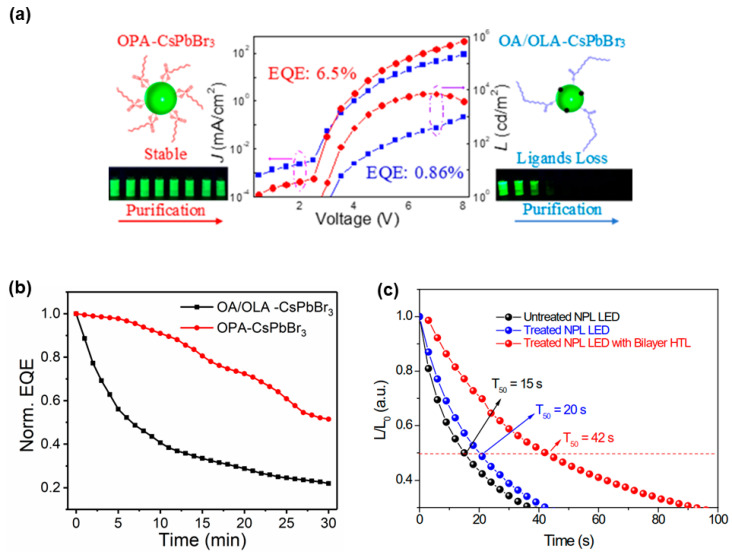
(**a**) Schematic illustration of the ligand treatment for high-performance PeLEDs. (**b**) Time-dependent EQE decay (under 2.5 mA cm^−2^) of PeLEDs based on OPA-CsPbBr_3_ and OA/OLA-CsPbBr_3_. Reproduced from reference [137] American Chemical Society, 2018. (**c**) Stabilities of PeLEDs with untreated nanoplatelet (NPL), treated NPL, and bilayer hole transport layer (HTL) (under 1 mA cm^−2^). Reproduced from reference [138], American Chemical Society, 2019.

In the case of device stability issue, Sun et al. replaced the OA and OLA ligands with octylphosphonic acid (OPA) for CsPbX_3_ nanocrystal emitters (Figure 3a) [137]. Owing to a strong interaction between OPA and lead atoms, the OPA-CsPbX_3_ preserved high PLQY (>90%) and high-quality dispersion in solvents after multiple purification processes. Additionally, the organic residue in purified OPA-CsPbBr_3_ was only ~4.6%, much lower than ~29.7% in OA/OLA-CsPbBr_3_. With the device architecture of ITO/PEDOT:PSS/poly-TPD/OPA-CsPbBr_3_ or OA/OLA-CsPbBr_3_/TPBi/LiF/Al, PeLEDs with OPA-CsPbBr_3_ showed a maximum EQE of 6.5% and improved device stability [137]. For example, the EQE of the OA/OLA-based LED decreased to ~20% of its original value, whereas the EQE of the OPA-based one remained >50% after 30 min (Figure 3b). Li et al. also adopted the surface ligand engineering strategy to enhance the PLQY and stability of blue perovskite nanoplatelet (NPL) by utilizing a short-chain halide ion-pair ligand DDAB [138]. The shorter-chain ligand treatment improved the electronic conductivity and charge injection of perovskite films, leading to PeLEDs with a peak EQE of 1.42% and enhanced device stability. For example, the T_50_ of treated CsPbBr_3_ NPL-based PeLEDs with the bilayer HTL reached 42 s (The device architecture was ITO/PEDOT:PSS/poly-TPD/CBP/CsPbBr_3_/TPBi/LiF/Al), which is much more stable than the untreated PeLEDs (Figure 3c). Recently, the dual ligands strategy (e.g., introducing two different ligands into perovskite precursor solution together) has also been demonstrated to be effective to enable PeLEDs with high efficiency and good device stability [139,140].

#### 3.2.3. Impurity Doping 

Impurity doping has been widely proved to be an efficient approach to afford nanocrystals with a variety of new electronic, optical, catalytic, transporting and magnetic characteristics [141,142,143]. With the insertion of atoms or ions of elements (e.g., transition metal, alkali metal) into host lattices, impurity-doped nanocrystals can possess desirable functionalities. Hence, impurity-doped nanocrystals are less sensitive than undoped ones to the chemical, thermal, and photochemical disturbances, considering that the self-quenching and reabsorption from enlarged Stokes shift can be eliminated [144,145,146]. Liu et al. took the first step to use the impurity doping strategy for perovskites by incorporating Mn ions into CsPbX_3_, demonstrating that the energy transfer between perovskites and Mn^2+^ had a crucial influence on the band-edge and Mn emissions [147]. Since then, a multiple of impurity doped perovskites have been reported.

Since impurity-doped nanocrystals can show not only the intrinsic merits of nanocrystals but additional advantages (e.g., enhanced thermal and chemical stability, improved PLQY, and reduced Auger recombination) [148,149,150], they are generally considered as promising candidate emitters for LEDs with high efficiency and increased stability. For the impurity-doped ABX_3_ perovskites, A-, B-, and X-site doping have been extensively applied to PeLEDs for high performance. Especially, A- or B-site doping can boost the device efficiency and stability by lowering the trap state and diminishing the nonradiative recombination, whereas X-site doping (e.g., the mixed halide systems Br/I and Cl/Br) is mainly used for tunable emissions [151,152,153]. 

**Figure 4:**
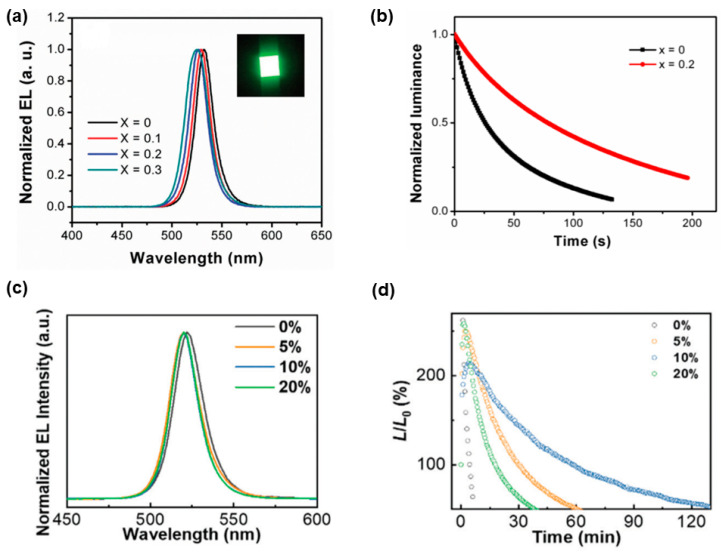
(**a**) EL spectra and photograph (under 4 V) for PeLEDs with different amounts of Cs doping and (**b**) Lifetime of PeLEDs with neat FAPbBr_3_ and mixed cation (Cs doping *x* = 0.2). Reproduced from reference [158], John Wiley and Sons, 2017. (**c**) EL spectra and (**d**) EL stability of PeLEDs based on the perovskite precursor solution incorporated with different amounts of MgBr_2_ (under a constant applied voltage). Reproduced from reference [167], Royal Society of Chemistry, 2016.

On one hand, A-site doping (i.e., the partial replacement of organic A-site cations (e.g., MA^+^, FA^+^) with alkali metal cations (e.g., Cs^+^, Rb^+^, K^+^, Na^+^)) can alleviate the inherent instability issue of organic-inorganic hybrid perovskites since organic cations are sensitive to oxygen, moisture, and temperature [154,155,156]. For instance, the ionic radius of Cs (1.81 Å) is smaller than that of FA (2.79 Å), indicating that Cs is more useful for the crystallization of the black phase of FA perovskites owing to the entropic stabilization [157]. Hence, alkali-metal-doped perovskites are possible to yield high-performance PeLEDs. Zhang et al. realized PeLEDs with mixed-cation FA_(1−x)_Cs_x_PbBr_3_ perovskites, in which FA^+^ was partially substituted with Cs^+^ during the synthesis process [158]. PeLEDs were fabricated with the device architecture of ITO/PEDOT:PSS/poly[(9,9-dioctylfluorenyl-2,7-diyl)-co-(4,4′-(N-(4secbutylphenyl)diphenylamine)] (TFB)/FA_(1−x)_Cs_x_PbBr_3_/TPBi/LiF/Al, showing 6.4-fold higher efficiency (10.09 cd A^−1^) and better stability relative to the FAPbBr_3_ PeLEDs (Figure 4a,b). In addition, other alkali metal cations doping has also been demonstrated to vastly improve the performance of PeLEDs [159]. Furthermore, other A-site doping strategies (e.g., Cs^+^ doped MA-based perovskites [160], MA^+^ doped FA-based perovskites [161], and FA^+^ doped Cs-based perovskites [162]) were also found to be effective to boost the performance of PeLEDs.

On the other hand, B-site doping (i.e., the partial substitution of Pb^2+^ with divalent (e.g., Mn^2+^, Ge^2+^, Sn^2+^, Cd^2+^, Co^2+^, Zn^2+^, Sr^2+^) or heterovalent cations (e.g., Ce^3+^, Sn^4+^)) can enhance the thermal and phase stability of perovskites [163,164,165]. Hence, high-performance PeLEDs can be built with the B-site doping strategy. For instance, Zou et al. stabilized CsPbBr_3_ lattice through Mn(II) substitution for air-stable PeLEDs, increasing the EQE from 0.81% for CsPbBr_3_ PeLEDs to 1.49% for Mn^2+^-doped CsPbBr_3_ PeLEDs as well as the device stability [166]. Huang et al. suppressed defect states in CsPbBr_3_ perovskite via Mg substitution, achieving PeLEDs with ~100-fold CE improvement (13.13 cd A^−1^) compared to undoped counterparts [167]. Besides the improved efficiency, Mg-doped PeLEDs displayed enhanced operation lifetime (Figure 4c,d). For example, PeLEDs fabricated with 10% MgBr_2_ in the precursor solution exhibit a 20-fold improvement in stability (138 min) measured at an initial luminance of ~100 cd m^−2^ under ambient conditions compared to pristine PeLEDs (7 min). The improved performance was attributed to the disappearance of the hole injection barrier due to the Mg substitution, balancing charge injection [168,169,170]. In fact, such working mechanism for the improved performance of PeLEDs with B-site doping strategy was also proposed by other groups [171]. For example, the maximum EQE of 5.92% was obtained for Sr^2+^-substituted based PeLEDs, which was 3-fold higher than that of unsubstituted PeLEDs [172].

#### 3.2.4. Precursor Solution Composition Optimization 

To broaden the prospect of PeLEDs for practical applications, the rapid degradation issue should be resolved. For perovskite thin films with the solution process, grain boundary related trap states and large crystal size usually lead to the poor performance of PeLEDs [173,174,175,176]. This is because trap states at adjacent grain boundaries can induce nonradiative energy transfer to shorten PL lifetime and lower PLQY, while large crystal size may impede the radiative recombination to decrease the EL performance [177,178,179,180,181]. Since smaller crystal size will form more grain boundaries and hence more trap states, the appropriate control of the grain boundary related trap states is required. Additionally, the ion migration in polycrystalline perovskite films crossing the grain boundaries during PeLED operation may induce nonradiative recombination, further reducing the device efficiency and stability [182,183,184]. Thus, the reduced trap state and small perovskite crystal size are significant to the performance of PeLEDs.

**Figure 5:**
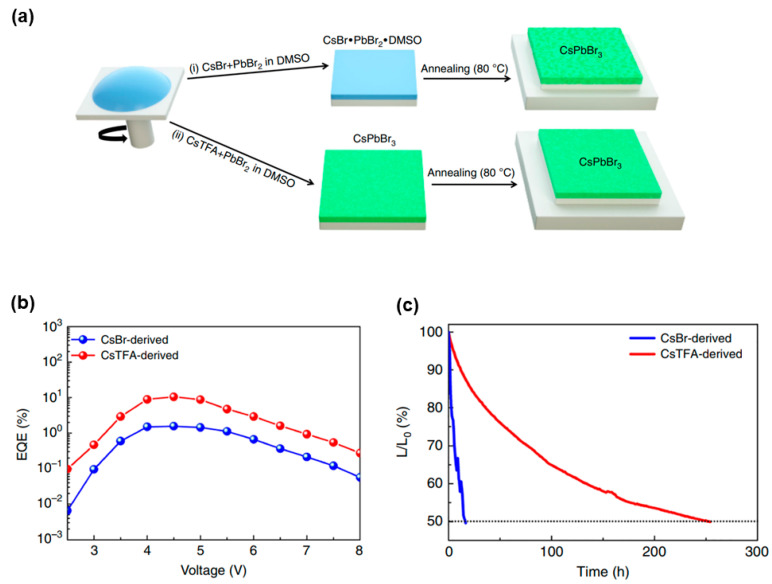
(**a**) Schematic presentation of the film fabrication procedure. Route (i) employed commonly used precursors CsBr and PbBr_2_, while the route (ii) employed CsTFA in place of CsBr. (**b**) EQE of the CsBr- and CsTFA-derived PeLEDs. (**c**) Lifetimes of PeLEDs based on CsBr(1.7)- and CsTFA(1.7)-derived CsPbBr_3_ perovskite films. Reproduced from reference [28], Springer Nature, 2019.

Toward the above-mentioned end, Yang et al. reported a fabrication method leading to dense, smooth, and pinhole-free CsPbBr_3_ films with high thermal stability, whose grain boundaries were well passivated for high-performance PeLEDs [28]. More specifically, CsPbBr_3_ films were obtained via one-step solution coating using cesium trifluoroacetate (TFA) as the cesium source instead of the conventional cesium bromide (CsBr), where the interaction of TFA^−^ anions with Pb^2+^ cations in the CsPbBr_3_ precursor solution greatly improved the crystallization rate of perovskite films (Figure 5). Compared to the CsBr route, the CsTFA-derived films showed a flatter energy landscape (a more homogeneous energy level distribution for charges), more stable crystal structure, better optical properties, and suppressed ion migration. By using a device architecture of ITO/PEDOT:PSS/perovskites/TPBi/LiF/Al, PeLEDs with TFA-derived mixed-cation FA_0.11_MA_0.10_Cs_0.79_PbBr_3_ showed a maximum EQE of 17%. Importantly, the CsTFA-derived PeLEDs showed a half-lifetime of over 250 h at an initial luminance of 100 cd m^−2^, which was ~17 times longer than that of CsBr-derived PeLEDs, indicating that efficient and stable PeLEDs could be developed through optimizing the grain boundaries from the perspective of the improvement of precursor composition.

### 3.3. The Innovation of Device Engineering 

After the selection of perovskite emitting materials, the device architecture is another key factor to decide the stability of PeLEDs. Since charge and exciton behaviors are varied with the device architecture, the innovation of device engineering becomes significant to guarantee the device performance [185,186,187,188,189]. To date, several efficient strategies have been utilized to innovate the device engineering of PeLEDs for the enhanced stability (e.g., modifying perovskite EMLs with thin insulating layers, balancing charge injection, exploiting inorganic charge transport layers, thin perovskite EMLs to increase the stability), which will be showed below.

#### 3.3.1. Modifying Perovskite EMLs with Thin Insulating Layers 

The as-prepared perovskite nanocrystals are restricted to be directly used in PeLEDs, since the low solubility of perovskites in solvents (e.g., toluene) generates poor films and ligands hampers the charge injection [190,191,192]. To overcome the bottleneck, the introduction of additional thin insulating layers to modify the perovskite EMLs is found to be an effective way to improve the performance of PeLEDs [193]. Such device design concept is previously reported in Cd-based QD-LEDs, where thin insulating layers (e.g., polymethylmethacrylate) are adopted to modify QD EMLs for the sake of high performance (e.g., high efficiency and long lifetime) [194].

**Figure 6:**
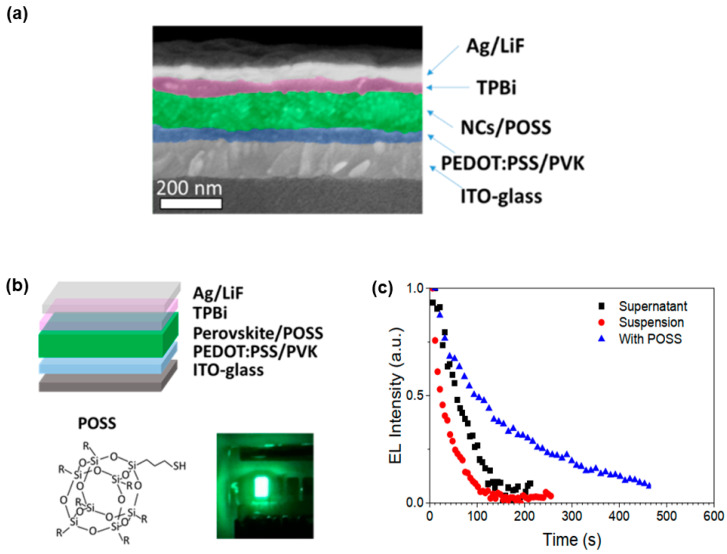
(**a**) Cross-sectional scanning electron microscope (SEM) image of PeLEDs. (**b**) Schematic illustration of the use of polyhedral oligomeric silsesquioxane (POSS) for high-performance PeLEDs. (**c**) EL stability measurements for different PeLEDs. Reproduced from reference [193], American Chemical Society, 2016.

Rogach and coworkers demonstrated that the use of an additive polyhedral oligomeric silsesquioxane (POSS) on CsPbBr_3_ could not only improve the solubility of perovskites but also contribute to balanced carrier conductivites in PeLEDs [193]. POSS was used due to the high chemical stability and optical transparency in the ultraviolet as well as visible spectral ranges. Additionally, POSS derivatives containing thiol groups could attach to perovskites and form uniform films [195]. The device architecture was ITO/PEDOT:PSS/poly(N-vinylcarbazole) (PVK)/CsPbBr_3_: POSS/POSS/TPBi/LiF/Ag, where the utilization of POSS as a solution additive improved the film-forming properties and POSS as an additional thin layer balanced the charge injection, as shown in Figure 6. On one hand, POSS improved the surface coverage and morphological features of the films deposited either from supernatant (concentration ~1 mg mL^−1^) or suspension (concentration ~50 mg mL^−1^) of perovskite nanocrystals. On the other hand, POSS acted as a hole-blocking layer to keep charges located within the EML for efficient recombination. As a result, the EQE of the resulting PeLEDs with POSS showed a 17-fold enhancement (0.35%) to the reference devices without POSS, while the lifetime of the resulting PeLEDs possessed 5-fold improvement to the reference devices. Then, other insulting layers (e.g., polyethylenimine ethoxylated (PEIE) [196]) have also been reported to modify the perovskite EMLs for the improvement of the efficiency and stability of PeLEDs.

#### 3.3.2. Balancing Charge Injection 

The charge injection and balance play a crucial role in the performance of LEDs. In particular, inefficient charge injection caused by interface barriers and/or ligands will easily result in Joule heat while poor charge balance will lead to charging emitters or Auger recombination, which deteriorates the device stability [196,197,198,199,200]. To resolve the issues of charge injection and balance in PeLEDs, the innovation of device engineering is essential. For example, Rogach and coworkers used the interface engineering strategy by introducing a thin film of perfluorinated ionomer (PFI) sandwiched between poly-TPD HTL and perovskite EML to improve the hole injection of CsPbBr_3_ PeLEDs, achieving 3-fold increase in peak luminance reaching 1377 cd m^−2^ [201]. Significantly, suitable ETLs are essential to high-performance PeLEDs, since charge transport, charge leakage, and charge balance can be largely affected by ETLs. 

**Figure 7:**
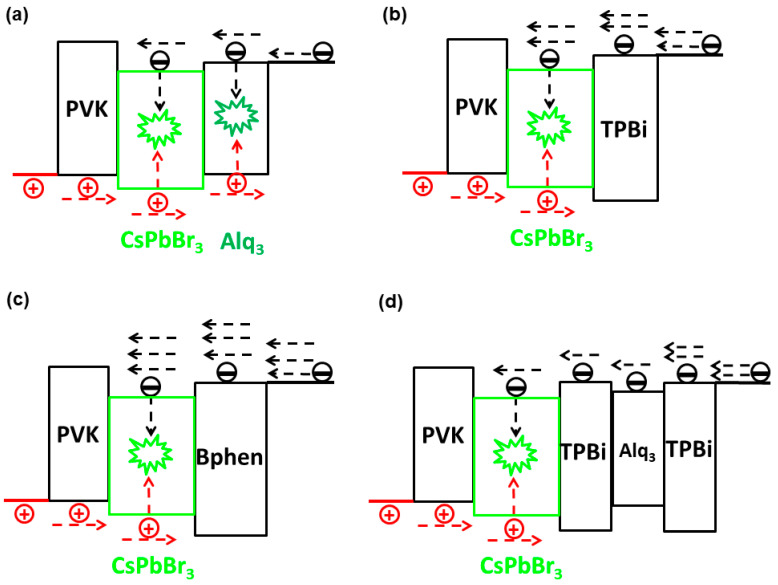
The working mechanisms of PeLEDs using different electron transport layers (ETLs): (**a**) Device G1, (**b**) Device G2, (**c**) Device G3, and (**d**) Device G4. Red and black arrows represented the hole and electron transport, respectively. Reproduced from reference [202], John Wiley and Sons, 2018.

Liu et al. investigated the effect of ETLs by using several types of ETLs [202]. The device architecture was ITO/PEDOT:PSS (40 nm)/PVK (10 nm)/CsPbBr_3_ (20 nm)/ETLs (35 nm)/Cs_2_CO_3_ (1 nm)/Al (100 nm), where ETLs were tris(8-hydroxyquinoline) aluminum (Alq_3_) for Device G1, TPBi for Device G2, 4,7-diphenyl-1,10-phenanthroline (Bphen) for Device G3, and TPBi/Alq_3_/TPBi for Device G4, as shown in Figure 7. The maximum EQE of PeLEDs with the ETL TPBi/Alq_3_/TPBi (1.43%) was 191% higher than that of the PeLED with the conventional ETL TPBi, while the stability of PeLEDs with the ETL TPBi/Alq_3_/TPBi was 307% longer than that of Device G2. This is because the electron mobility of Alq_3_ (1.4 × 10^−6^ cm^2^ V^−1^ s^−1^) is almost equal to the hole mobility of PVK (1.0 × 10^−6^ cm^2^ V^−1^ s^−1^) [203], while the electron mobility of TPBi (3.3 × 10^−5^ cm^2^ V^−1^ s^−1^) and Bphen (3.9 × 10^−4^ cm^2^ V^−1^ s^−1^) is much higher than the hole mobility of PVK [204]. Thus, the insertion of Alq_3_ impeded the electron transport, achieving better charge balance in Device G4. Therefore, the better charge balance enhanced the stability.

#### 3.3.3. Exploiting Inorganic Charge Transport Layers

**Figure 8:**
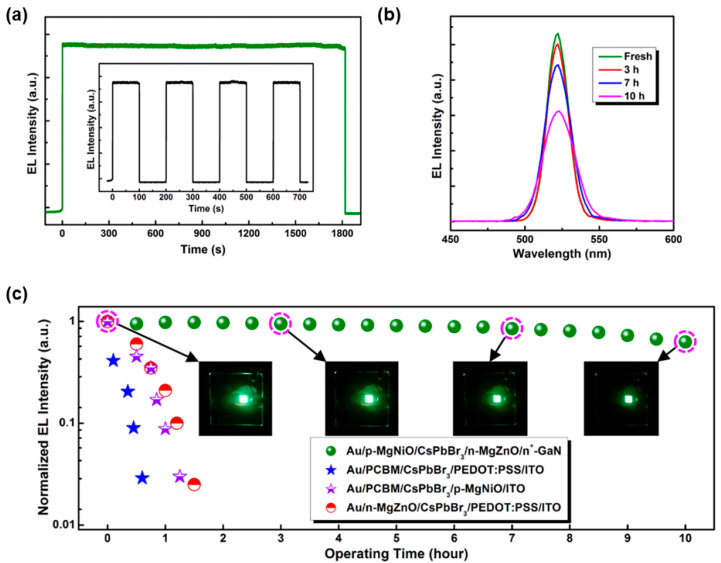
(**a**) Time dependence (30 min) of the room temperature EL intensity of the PeLED under a dc bias of 8.0 V. The inset showed the responses of the EL intensity for four switch-on/switch-off pulses at 8.0 V. (**b**) EL spectra obtained after different running periods. (**c**) PeLEDs as a function of running time (under 10.0 V). The insets showed the corresponding photographs of PeLED after different running periods. Reproduced from reference [211], American Chemical Society, 2017.

In general, the inherent chemical stability of organic charge transport materials is poorer than that of the inorganic counterparts. Thus, the exploitation of inorganic charge transport layers will be an effective strategy to enhance the stability of LEDs [205,206,207]. In OLEDs and Cd-based QD-LEDs, various types of inorganic materials have been explored to replace conventional conducting polymer or small molecules (e.g., PEDOT:PSS, TPBi) for the stability [208,209,210]. To improve the lifetime of PeLEDs, Shi et al. used n-MgZnO and p-MgNiO as the inorganic ETL and HTL, respectively [211]. The optimized device architecture (Device I) was c-Al_2_O_3_ (substrate)/n^+^-GaN (2 μm)/n-Mg_0.38_Zn_0.62_O (45 nm)/CsPbBr_3_ (55 nm)/p-Mg_0.23_Ni_0.77_O (80 nm)/Au (30 nm), where patterned low-resistance n^+^-GaN was the electron source, conducting template, and a transparent window. The resulting PeLED demonstrated a maximum EQE of 2.39% and a significantly improved operation stability (Figure 8). For example, Figure 8a inset showed the responses of the EL intensity for 4 switch-on/switch-off pulses at 8.0 V, and no degradation on the device performance, indicating the good stability. Additionally, the unencapsulated PeLEDs under ambient air condition (28 °C, 30–50% humidity) in a continuous bias of 10.0 V could operate continuously for 10 h with an emission decay of ∼20%. By replacing MgZnO/MgNiO with organic charge transport layers PCBM/PEDOT:PSS, the device stability was seriously degraded, suggesting the advantage of inorganic materials for the lifetime [212]. 

With the strategy of exploiting inorganic charge transport layers, Tan et al. used the solution-processed ZnMgO as the ETL for the improved performance [213]. In addition, Li-doped TiO_2_ were also found to be an efficient inorganic ETL for PeLEDs [214,215]. On the other hand, the replacement of PEDOT:PSS with NiOx as the hole injection and transport layer was demonstrated to be an excellent way for PeLEDs with enhanced lifetime [216,217,218].

#### 3.3.4. Thin Perovskite EMLs to Increase the Stability 

The thickness of each layer in LEDs has a great influence on the device performance, especially for the thickness of EMLs [219,220,221]. This is because charge and exciton behaviors (e.g., exciton generation and recombination) will be correspondingly changed according to the thickness of EMLs [222,223,224]. In OLEDs, Liu et al. studied the effect of the thickness of EMLs and found that thin EMLs could affect the efficiency, voltage, luminance, and stability [225]. In Cd-based QD-LEDs, it is also demonstrated that the device performance can be affected by the thickness of EMLs [226]. In the case of PeLEDs, the influence of EML thickness is still somewhat unclear since the thickness of perovskite EMLs considerably changes from <20 nm to >200 nm [227,228].

**Figure 9:**
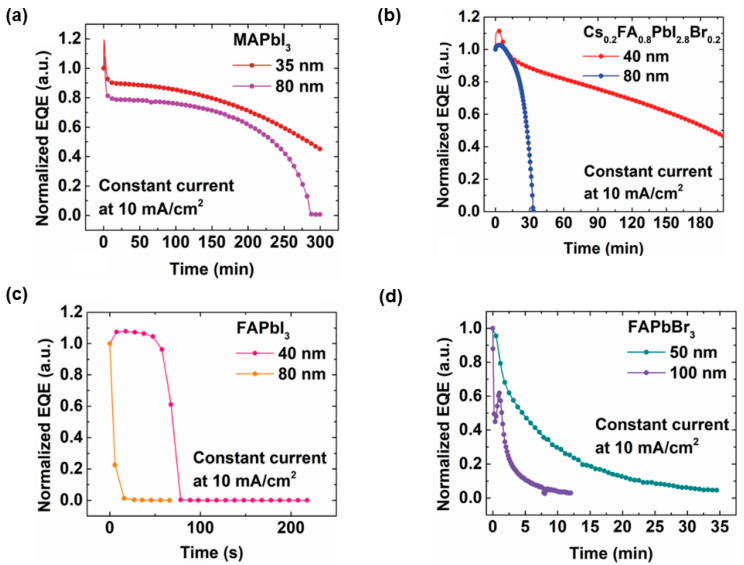
Operational stability of PeLEDs at 10 mA cm^−2^ for (**a**) MAPbI_3_, (**b**) Cs_0.2_FA_0.8_PbI_2.8_Br_0.2_, (**c**) FAPbI_3_, and (**d**) FAPbBr_3_ with various thicknesses. Reproduced from reference [229], John Wiley and Sons, 2018.

To comprehend the effect of EML thickness, Zhao et al. demonstrated that thin perovskite EMLs in the range of 35–40 nm was crucial for the lifetime as well as efficiency [229]. Their device architecture was ITO (150 nm)/poly-TPD (poly[*N*,*Nʹ*-bis(4-butylphenyl)-*N*,*N*ʹ-bis(phenyl)-benzidine] 25 nm)/EMLs/TPBi (40 nm)/LiF (1.2 nm)/Al (100 nm), where EMLs were MAPbI_3_, Cs_0.2_FA_0.8_PbI_2.8_Br_0.2_, FAPbI_3_, and FAPbBr_3_, as shown in Figure 9. By systematically investigating multiple thicknesses of EMLs, it was found that the EML thickness of 35–40 nm for all perovskite compositions was ideal to the operational stability. The underlying reason for the increased stability was the reduced Joule heating. Particularly, thick perovskite EMLs were related to high junction temperatures in PeLEDs. This was because: (i) the ultralow thermal conductivity of MAPbI_3_ (0.3 W m^−1^ K^−1^) rendered that the thermal dissipation was inefficient for thick EMLs, (ii) a larger portion of the input power was converted to heat since the initial EQE of thicker PeLEDs was lower, (iii) thicker PeLEDs showed faster degradation, forming positive feedback with heat generation/accumulation. As a consequence, the reduced Joule heating in turn suppressed thermally activated ionic processes [230], improving the stability. Meanwhile, thin perovskite EMLs were beneficial to improve light outcoupling and hence EQE. For example, maximum EQEs of 17.6% for Cs_0.2_FA_0.8_PbI_2.8_Br_0.2_, 14.3% for MAPbI_3_, 10.1% for FAPbI_3_, and 11.3% for FAPbBr_3_-PeLEDs were realized [229]. 

### 3.4. The Manipulation of Optical Effects

To date, a number of schemes have been reported to increase the performance of PeLEDs and the EQE can surpass 20%. To further boost the EQE and stability, the manipulation of optical effects in PeLEDs is needed. Since the N of perovskite EMLs is higher than that of organic transport layers, optical losses of >70% of emitted photons occur during the operation. Therefore, the maximum EQE of PeLEDs is limited by outcoupling efficiency and restricted to be around 20%, with the remainder of light being trapped within the thin film and substrate materials, or parasitic absorption [231,232,233]. Thus, the study of device architectures that can enhance the outcoupling efficiency is required.

**Figure 10:**
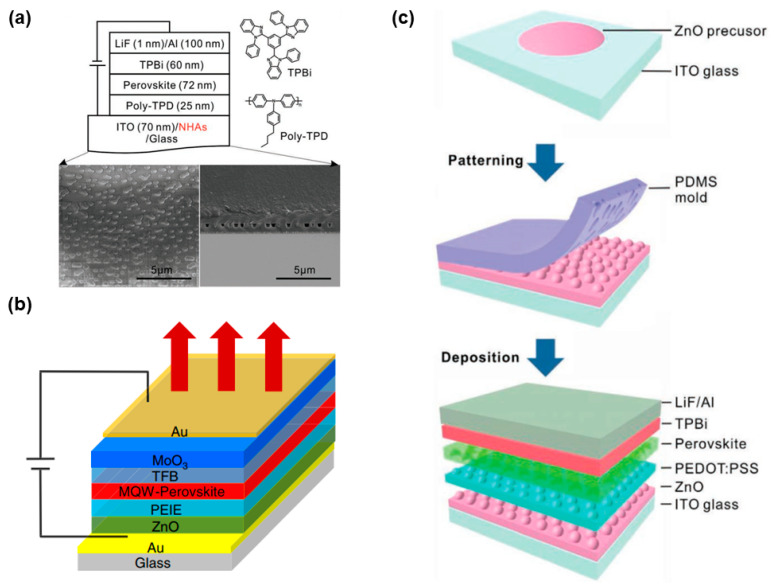
(**a**) Device structures of PeLEDs with and without nanohole arrays (NHAs), the molecular structure of organic transporting layers, and SEM images of the structure. Reproduced from reference [237], John Wiley and Sons, 2019. (**b**) Structure of the PeLEDs. A thick gold film was used as the total-reflection bottom electrode, and a thin gold film was used as the semitransparent top electrode, forming a Fabry-Perot microcavity. Reproduced from reference [240], Springer Nature, 2020. (**c**) Schematic illustration of the fabrication process of a PeLED with imprinted nanostructures. Reproduced from reference [91], John Wiley and Sons, 2019.

Some effective approaches have been demonstrated to substantially enhance the performance of PeLEDs via the manipulation of optical effects (e.g., the internal or external light outcoupling methods) [234,235,236]. Despite these approaches were mainly focused on the EQE enhancement, it is believed that the device stability can be also improved through the introduction of light outcoupling technology. For the internal light outcoupling methods, Lee et al. developed MAPbI_3_ PeLEDs using a randomly distributed nanohole array (NHA) embedded in a SiN layer between ITO anode and glass substrate (Figure 10a), where SiN with a N of 2.02 at the peak emission possessed a high-index contrast with the voids of the NHA with N of 1.0 [237]. This layer compensated for the high N of the perovskites and aids outcoupling of waveguided and substrate modes, leading to PeLEDs with NHAs having 1.64 times higher light extraction (14.6% EQE) than PeLEDs without NHAs.

Another internal light outcoupling method is the use of the microcavity effect, since the radiative decay rate can be increased through the Purcell effect [238] when placing an emitter in a Fabry-Perot microcavity [239]. Wang et al. employed the microcavity effect to enhance light extraction of PeLEDs [240]. The microcavity was formed via a total-reflection Au bottom electrode and a semitransparent Au top electrode in a top-emission architecture, where the length of the cavity was tuned by changing the thickness of carrier transport layers. The device architecture was optimized to be glass/Au (100 nm)/PEIE modified ZnO (37 nm)/perovskite (~35 nm)/TFB (76 nm)/molybdenum oxide (MoO_3_, 7 nm)/Au (15 nm), as shown in Figure 10b. As a consequence, PeLEDs with an EQE of 20.2% and a radiant exitance of 114.9 mW cm^−2^ were yielded.

In order to remarkably boost the light extraction efficiency of PeLEDs, both internal outcoupling methods and external outcoupling approaches should be simultaneously adopted. Tang et al. demonstrated such strategy by using bioinspired moth-eye nanostructures at the front electrode/perovskite interface as the internal outcoupling method (Figure 10c), where the EQE was improved from 13.4% (the reference flat PeLEDs) to 20.3% due to the enhanced outcoupling of the waveguided light into the substrate mode [91]. Then, they mounted a half-ball lens on top of the glass substrate with an index matching gel as the external outcoupling approach to extract the light trapped in the substrate mode. As a result, the maximum EQE PeLEDs was up to 28.2%, which is the highest efficiency for PeLEDs. 

### 3.5. The Introduction of Advanced Encapsulations

Advanced encapsulations are critical to the stability of PeLEDs, since perovskites and organic transport materials are sensitive to the environmental factors. As the device architectures of PeLEDs can be classified into bottom-emitting, top-emitting, and transparent types, the encapsulation technology is varied among these three types [241,242,243]. In the case of bottom-emitting PeLEDs, the most widely used approach is the utilization of UV-cured epoxy and cover glass together with a desiccant inside devices. However, such an approach is not perfect for top-emitting or transparent PeLEDs, since the light in these devices will be emitted through the encapsulating layer into the air. As a result, the total reflection occurs at the interface will reduce the light extraction efficiency because of the different N between the adjacent layers [244,245,246]. Therefore, the enhancement of light extraction efficiency should be considered for the encapsulation technology of top-emitting and transparent PeLEDs. In addition, the spectral stability can be affected by the encapsulating layer.

Despite top-emitting or transparent PeLEDs has been rarely demonstrated so far, these two types of PeLEDs are believed to be significant for the new-generation display and lighting technology [247]. For the future development of top-emitting and transparent PeLEDs, the introduction of the encapsulation experience from other kinds of LEDs are helpful. For example, Chen et al. showed a high-gravity-hydrolysis approach for the synthesis of transparent nanozirconia/silicone hybrid materials, which was used as the encapsulation of GaN-based white LEDs with enhanced light extraction efficiency and reduced blue light exposure [248]. These reported encapsulation methods are also suitable for PeLEDs.

On the other hand, thin-film encapsulation technique is a promising candidate for the future development of PeLEDs, particularly for flexible PeLEDs since a principal limitation of flexible LEDs is the lifetime. The thin-film encapsulations consist of multilayer barrier coatings to avoid the moisture and oxygen, where the multilayers are usually formed by the alternating layers of inorganic and organic layers or alternating different inorganic layers. For example, an effective thin-film encapsulation was composed of Al_2_O_3_ layers between polyacrylate layers [249], where Al_2_O_3_ was diffusion barriers to water/oxygen while the polymer layers decoupled defects in the oxide as well as allowed for flexibility [250,251]. Nanolaminate structures composed of alternating Al_2_O_3_ and ZrO_2_ sublayers grown by atomic layer deposition at 80 °C were used to realize stable OLEDs [252], while the cathode encapsulation of OLEDs by Al_2_O_3_ films and Al_2_O_3_/a-SiNx:H stacks could achieve a water vapor transmission rate of ≤2 × 10^−6^ g m^−2^ day^−1^ and 4 × 10^−6^ g m^−2^ day^−1^ (20 °C/50% relative humidity) for 20–40 nm Al_2_O_3_ and 300 nm a-SiNx:H films, respectively [253]. Since the first flexible PeLED was realized in 2015 [254], an increasing attention has been paid to improve the performance of flexible PeLEDs [255,256,257]. As a consequence, thin-film encapsulation technique is anticipated to be a crucial candidate for the flexible encapsulations of PeLEDs.

## 4. Summary and Outlook 

As the peak EQE of PeLEDs has been demonstrated to exceed 20% to date, the next critical limitation of real commercialization is the device lifetime. Fortunately, increasing attention is being paid to comprehend the issue of device stability, leading to the flourishing development of PeLEDs possessing improved lifetime. Meanwhile, the understanding of the prolonged lifetime is also beneficial to enhance other parameters of LEDs (e.g., enhanced efficiency/luminance, and lowered operational voltage) [258,259,260,261]. With the intensive endeavors, PeLEDs are highly promising for the future-generation displays, lighting, and signaling. In this review, we have mainly focused on the recent advances in the realization of PeLEDs with improved lifetime. In particular, we have emphasized various representative strategies to boost the device stability, including (i) the design of perovskite emitting materials (e.g., anti-solvent engineering, surface ligand engineering, impurity doping, and precursor solution composition optimization), (ii) the innovation of device engineering (e.g., modifying perovskite EMLs with thin insulating layers, balancing charge injection, exploiting inorganic charge transport layers, thin perovskite EMLs to increase the stability), (iii) the manipulation of optical effects, and (iv) the introduction of advanced encapsulations. More specific performance of PeLEDs possessing improved lifetime have been concluded in Table 1. 

PeLEDs have been demonstrated to possess outstanding performance, however, some serious issues still exist, particularly for the device lifetime issue. First, despite the lifetime of PeLEDs is step-by-step increased, it is far away from the required practical standard (e.g., the lifetime of ≥100,000 h at ≥100 cd m^−2^ for displays and ≥10,000 h at ≥1000 cd m^−2^ for the solid-state lighting). Therefore, much more efforts are needed to be made for the long device lifetime. In addition, the reported PeLEDs with improved lifetime usually contain Pb atoms, which restricts the extensive applications of PeLEDs [262,263,264]. Furthermore, the lifetime of blue PeLEDs is scarcely documented, restricting the general full-color applications. Moreover, almost no attention has been paid to the lifetime of white PeLEDs. 

To further prolong the lifetime of PeLEDs, several research directions may be crucial in the near future. (i) The introduction of state-of-the-art concepts from OLEDs, Cd-based QD-LED, and even III-Nitride based LEDs (e.g., solving critical challenges related to material quality, light extraction, and IQE) [265,266,267] will accelerate the development of highly stable PeLEDs. (ii) Much more attention is required for heavy-metal-free PeLEDs, or else the toxicity issue will impede PeLEDs to enter the mainstream display, lighting, and signaling markets [268,269,270]. (iii) Endeavors are urgent to be taken to the investigation of blue and white PeLEDs, otherwise PeLEDs will still lag far behind other types of LEDs [271,272,273]. (iv) Tandem PeLEDs are necessary to be explored, since tandem device architectures can amazingly boost the lifetime [274,275,276]. (v) Since the emission mechanism of PeLEDs is still somewhat controversial, the deep comprehending of charge and exiciton behaviors is necessary to be studied [277,278,279]. Although the pursuing commercial products still faces a number of challenging tasks, PeLEDs with satisfactory operational stability are anticipated in the near future via the gradual understanding of the insight of perovskite emitting materials, device engineering, optical effects, and advanced encapsulations. Furthermore, the development of PeLEDs possessing improved lifetime will shed light on the other optoelectronic applications (e.g., lasers, solar cells, and sensors) [280,281,282,283,284].

## Figures and Tables

**Table 1:** Summarized performances for representative PeLEDs possessing improved lifetime.

Emitters ^a^	V_on_^b^ (V)	EQE_max_^c^ (%)	L_max_^d^ (cd m^−2^)	Lifetime ^e^	Reference
CsTFA-derived CsPbBr_3_	2.8	10.5	16,436	T_50_ = 250 h at 100 cd m^−2^	[28]
CsPbBr_3_-PEO-CF	2.6	4.76	51,890	82% of the initial efficiency after 80 h	[124]
OPA-CsPbBr_3_	2.8	6.5	7085	>50% of the initial efficiency after 30 min	[137]
DDAB-CsPbBr_3_ NPLs	3.6	1.42	41.8	T_50_ ≈ 42 s at 1 mA cm^−2^	[138]
FA_0.8_Cs_0.2_PbBr_3_	3.5	2.8	55,005	T_50_ ≈ 85 s	[158]
CsPb_0.9_Mg_0.1_Br_3_	~2.7	3.6	25,450	T_50_ = 138 min at ~100 cd m^−2^	[167]
CsPbBr_3_	5.8	0.35	2983	T_50_ ≈ 120 s at 7 V	[193]
CsPbBr_3_	4.8	1.43	452	T_50_ = 460 s at 9 V	[202]
CsPbBr_3_	3.0	2.39	3809	~80% of the initial efficiency after 10 h	[211]
FAPbBr_3_	-	11.3	79,700	T_50_ ≈ 6 min at 10 mA cm^−2^	[229]

^a^ The employed emitters in PeLEDs. ^b^ Turn-on voltage. ^c^ Maximum EQE. ^d^ Maximum luminance. ^e^ The lifetime of PeLEDs.

## Data Availability

Data available in a publicly accessible repository.
